# Natural Products for the Potential Use of Neuroprotective and Neurorestorative Effects in Stroke

**DOI:** 10.3390/ph16111516

**Published:** 2023-10-25

**Authors:** Hansen Chen, Qingkun Liu

**Affiliations:** 1 Department of Neurosurgery, School of Medicine, Stanford University, Stanford, CA 94305, USA; 2 Department of Neuroscience, Icahn School of Medicine at Mount Sinai, New York, NY 10029, USA

Stroke is the second leading cause of death and the third leading cause of disability worldwide, with limited treatment options. Emerging research illuminates the potential of natural products to mitigate stroke-induced damage and promote stroke recovery. Contained within this Special Issue are the latest findings and perspectives concerning the utilization of natural products in stroke treatment. This compilation encompasses investigations into their effects, encompassing neuroprotection, neurogenesis, and the enhancement of stroke outcomes. Provided herein is a succinct summary of these noteworthy studies.

**Anethole’s Neuroprotective Effects and Its Mechanism**: In their study, Younis and Mohamed reported that pretreatment of Anethole, one of the major constituents of several plant oils, improved ischemic outcomes. Specifically, they investigated the impact on infarct volume, brain water content, and neurological score in a Sprague Dawley male rat MCAo model at a dosage of 125 or 250 mg/kg. Mechanistically, Anethole may protect against stroke by boosting blood–brain barrier integrity via modulating Matrix-metalloproteinases and diminishing oxidative stress, inflammation, and apoptosis through the JNK/p38 pathway [1].

**Geopung–Chunghyuldan’s Neuroprotective Potential: Evidence from In Vitro and In Vivo Stroke Models:** In addition, an original research article by Tae-Hoon Park et al. demonstrated that Geopung–Chunghyuldan (GCD), a combination of Chunghyuldan (CD, a natural product from Korea medicine) and cardiotonic pills, has a neuroprotective effect on in vitro oxygen–glucose deprivation and in vivo mice model of permanent middle cerebral artery occlusion. In particular, GCD significantly reduced SH-SY5Y cell death induced by oxygen–glucose deprivation and attenuated infarct volume in the pMCAo mice model [2]. Notably, GCD also exerts better protection against cell death in vitro and neuronal damage in vivo in a stroke model than CD alone, indicating GCD’s pharmaceutical potential in stroke treatment.

**BBF’s Neuroprotective Effect in Stroke**: Jingyang Le et al. explored the neuroprotective effects of Brilliant Blue FCF (BBF), a synthetic organic compound used as a food pigment, in a rat middle cerebral artery occlusion (MCAO) stroke model. BBF treatment reduced brain infarct size and mitigated brain edema, improving neurological outcomes. Both in vitro and in vivo data suggest that BBF’s neuroprotection may be linked to the inhibition of ERK and/or GSK-3β pathways [3].

**Exosome Therapy for Stroke Protection:** Exosomes, derived from various cell sources, such as mesenchymal stem cells, have shown the potential to enhance stroke outcomes in preclinical studies [4,5,6]. In this collection, Jin Sun et al. investigated brain microvascular endothelial cell (BMVEC) exosomes’ potential to shield neurons from ischemic stroke damage. Treatment involving tail vein injection of these exosomes improved neuromotor function, reduced neuronal apoptosis, mitigated synaptic damage, and increased the cerebral blood flow of stroke-affected mice. The in vitro data consistently demonstrated the neuroprotective effects of BMVEC-derived exosomes under oxygen and glucose deprivation (OGD). This study suggests that BMVEC-derived exosomes are a promising avenue for ischemic stroke treatment, prompting further research into their underlying mechanisms and bioactive cargo [7].

**Addressing Post-Stroke Depression with a Chinese Herb Compound**: Post-stroke depression (PSD) affects about one-third of stroke patients, negatively impacting functional outcomes and quality of life [8]. The available treatments for PSD are currently limited. In this collection, Xi Chen et al. presented Astragaloside VI (AsVI) from the Chinese Medicine herb Radix Astragali as a potential treatment for post-stroke depression. AsVI treatment reduced depression-like behaviors in rats with PSD and prevented CORT-induced apoptotic cell death in neuronal PC-12 cells. AsVI treatment reversed the decline in dopamine (DA) and serotonin (5-HT) levels in both PSD rat brains and CORT-induced PC-12 cells, as well as upregulated the NRG-1-mediated MEK/ERK pathway, which is associated with improved PSD [9].

**A Review on Chinese Medicine’s Stroke Recovery Potential**: Chinese herbs and their derived compounds hold promise in neuroprotection and stimulating neurogenesis [10,11]. Lin Li et al. summarized recent updates on Chinese Medicine’s role in boosting neurogenesis in preclinical stroke studies. Notable herbs, such as Radix Astragali, Ginseng, Panax pseudoginseng, Salvia miltiorrhiza Bge, and Momordica charantia, and their bioactive compounds have enhanced neurogenesis in animal stroke models. The authors emphasized the need for robust evidence to demonstrate the efficacy of these compounds and Chinese Medicine. Enhancing technologies such as high-throughput screening, small-molecule probes, label-free detection, and target validation are essential for thoroughly analyzing Chinese medicine-regulated signaling pathways and their regulatory processes [12].

In conclusion, this Special Issue underscores the potential of natural products in protecting against ischemic stroke injury and expediting stroke recovery. These studies elucidate hope by blending time-honored wisdom with modern research.

## References

[1] Younis N.S., Mohamed M.E. (2023). Anethole Pretreatment Modulates Cerebral Ischemia/Reperfusion: The Role of JNK, P38, MMP-2 and MMP-9 Pathways. Pharmaceuticals.

[2] Park T.-H., Lee H.-G., Cho S.-Y., Park S.-U., Jung W.-S., Park J.-M., Ko C.-N., Cho K.-H., Kwon S., Moon S.-K. (2023). A Comparative Study on the Neuroprotective Effect of Geopung-Chunghyuldan on In Vitro Oxygen-Glucose Deprivation and In Vivo Permanent Middle Cerebral Artery Occlusion Models. Pharmaceuticals.

[3] Le J., Xiao X., Zhang D., Feng Y., Wu Z., Mao Y., Mou C., Xie Y., Chen X., Liu H. (2022). Neuroprotective Effects of an Edible Pigment Brilliant Blue FCF against Behavioral Abnormity in MCAO Rats. Pharmaceuticals.

[4] Song Y., Shi R., Liu Y., Cui F., Han L., Wang C., Chen T., Li Z., Zhang Z., Tang Y. (2023). M2 Microglia Extracellular Vesicle MiR-124 Regulates Neural Stem Cell Differentiation in Ischemic Stroke via AAK1/NOTCH. Stroke.

[5] Wang C., Börger V., Mohamud Yusuf A., Tertel T., Stambouli O., Murke F., Freund N., Kleinschnitz C., Herz J., Gunzer M. (2022). Postischemic Neuroprotection Associated with Anti-Inflammatory Effects by Mesenchymal Stromal Cell-Derived Small Extracellular Vesicles in Aged Mice. Stroke.

[6] Zhang Z.G., Buller B., Chopp M. (2019). Exosomes—beyond Stem Cells for Restorative Therapy in Stroke and Neurological Injury. Nat. Rev. Neurol..

[7] Sun J., Yuan Q., Guo L., Xiao G., Zhang T., Liang B., Yao R., Zhu Y., Li Y., Hu L. (2022). Brain Microvascular Endothelial Cell-Derived Exosomes Protect Neurons from Ischemia–Reperfusion Injury in Mice. Pharmaceuticals.

[8] Poststroke Depression: A Scientific Statement for Healthcare Professionals from the American Heart Association/American Stroke Association—PubMed. https://pubmed.ncbi.nlm.nih.gov/27932603/.

[9] Chen X., Shen J., Zhou Q., Jin X., Liu H., Gao R. (2022). *Astragaloside* VI Ameliorates Post-Stroke Depression via Upregulating the NRG-1-Mediated MEK/ERK Pathway. Pharmaceuticals.

[10] Gao C., Shen J. (2017). Metabolic Factors and Adult Neurogenesis: Impacts of Chinese Herbal Medicine on Brain Repair in Neurological Diseases. Int. Rev. Neurobiol..

[11] Zhang E., Shen J., So K.F. (2014). Chinese Traditional Medicine and Adult Neurogenesis in the Hippocampus. J. Tradit. Complement. Med..

[12] Li L., Li X., Han R., Wu M., Ma Y., Chen Y., Zhang H., Li Y. (2023). Therapeutic Potential of Chinese Medicine for Endogenous Neurogenesis: A Promising Candidate for Stroke Treatment. Pharmaceuticals.

